# Polarization-Entangled Photon Pairs From Periodically-Poled Crystalline Waveguides Over a Range of Frequencies

**DOI:** 10.6028/jres.118.018

**Published:** 2013-08-15

**Authors:** Dylan A Heberle, Zachary H Levine

**Affiliations:** 1 Rochester Institute of Technology, 1 Lomb Memorial Drive, Rochester, NY 14623-5603; 2 National Institute of Standards and Technology, Gaithersburg, MD 20899-8441

**Keywords:** entangled photons, frequency and angle tuning, periodically-poled waveguide, Rb:KTP (rubium doped potassium titanyl phosphate)

## Abstract

We propose a method to extend the frequency range of polarization entanglement in periodically poled rubidium-doped potassium titanyl phosphate (Rb:KTP) waveguides. Our calculations predict that output wavelengths from 1130 nm to 1257 nm may be achieved using Rb:KTP by the appropriate selection of a direction of propagation for the waveguide. The fidelity using a poling period of 1 mm is approximately 0.98.

## 1. Introduction

Photon entanglement is a requirement for various applications in nonlinear optics and quantum mechanics [1,2]. Polarization entanglement has been implemented in areas such as quantum teleportation [3], quantum informatics [4], and quantum cryptography [5]. Recently, entangled states have been used in the design of quantum circuitry using domain-engineering [6,7]. Another recent study demonstrated that high spatial entanglement was possible using nonlinear waveguide arrays [8].

Other studies have focused on the development of entangled photon pairs using periodically poled crystals and waveguides. One of the most commonly poled materials is potassium titanyl phosphate (KTP). Kim *et al.* [9] placed periodically poled KTP (PPKTP) inside a Sagnac interferometer to produce entangled photon pairs. Periodically poled lithium niobate (PPLN) has been used to achieve polarization-entangled photon pairs emitted at the telecom wavelength [10] and to demonstrate the steering of entangled photons [6]. Recently, the generation of entangled photons directly from an optical fiber has been demonstrated, albeit with low source intensity [11].

Our group proposed a method [12] which, like Ref. [11], allows for the direct generation of polarization-entangled photon pairs. However, the proposed method was restricted to operation at very few frequencies. To realize a range of quasi-phase-matched operational frequencies for type-II spontaneous parametric downconversion (SPDC) in periodically poled waveguides, we propose orienting the waveguide at an angle relative to the crystalline structure during fabrication. As different orientations of the waveguide are selected, the effective indices of refraction are changed, allowing a range of frequencies to be quasi-phase matched. We take advantage of the biaxial nature of KTP to permit this reorientation. Although the fabrication of PPKTP waveguides at an arbitrary orientation does not appear to have been achieved, lateral patterning of periodically poled lithium niobate [13] and lithium tantalate [6] waveguides have been realized experimentally.

## 2. Theoretical Development

Polarization entanglement can be achieved using type-II SPDC in periodically poled waveguides. In this process, a high-frequency photon, called the pump photon, is downconverted into two lower frequency photons, known as the signal and idler. The pump photon and either the signal or the idler photon are polarized in the same direction which is orthogonal to the polarization of the third photon. In the previous study [12], periodic poling was used to achieve quasi-phase matching for two different type-II SPDC processes simultaneously. The poling period Λ was used to offset the wave vector mismatch ∆*k* given by

(1) $$\Delta k_{m}\;(\omega_{s},\omega_{i})=k_{v}(\omega_{s})+k_{H}(\omega_{i})-k_{H}(\omega_{p})+\frac{2\pi m}{\Lambda }$$

where *ω_p_*, *ω_s_*, and *ω_i_* are the angular frequencies of the pump, signal, and idler photons respectively and *V and H* stand for the vertical and horizontal polarizations. A polarization-entangled state was achieved by quasi-phase matching the *m* = ±1 modes, as shown by the following equations

$$\Delta k_{1}\;(\omega_{s},\omega_{i})=k_{v}(\omega_{s})+k_{H}(\omega_{i})-k_{H}(\omega_{p})+\frac{2\pi }{\Lambda }=0$$

(2) $$\Delta k_{-1}\;(\omega_{i},\omega_{s})=k_{v}(\omega_{i})+k_{H}(\omega_{s})-k_{H}(\omega_{p})-\frac{2\pi }{\Lambda }=0.$$

Our scheme uses the same entanglement mechanism but proposes a selection of the waveguide orientation during fabrication to achieve a range of operational frequencies.

For bulk KTP and Rb:KTP waveguides, the crystal *Z* axis needs to be aligned normal to the surface so that the standard diffusion process may occur. However, we consider propagation directions of the waveguide at an angle *ϕ* in the crystal *XY* plane, with *ϕ* = 0 corresponding to propagation along the *X*-axis.

The indices of refraction for KTP and Rb:KTP are calculated from the Sellmeier equations of König and Wong [14], who introduce a new Sellmeier equation for $n_{Y}^{\left(KTP\right)}$ and suggest the Sellmeier equation of Fradkin *et al.* [15] for $n_{Z}^{\left(KTP\right)}$. To find the difference between the indices of KTP and RTP (rubidium titanyl phosphate) for the waveguides, we use the Sellmeier equations from Cheng *et al.* [16] who report the values for both crystals using one set of instruments. In this study, we introduce a new Sellmeier equation for $n_{X}^{\left(KTP\right)}$ given by

(3) $$n_{X}^{\left(KTP\right)}=n_{Y}^{\left(KTP,König\right)}-n_{Y}^{\left(KTP,Cheng\right)}+n_{X}^{\left(KTP,Cheng\right)}.$$

This equation is formulated to approximate the value of $n_{X}^{\left(KTP\right)}$ so that it agrees with the values of $n_{Y}^{\left(KTP\right)}$ and $n_{Z}^{\left(KTP\right)}$ from König and Wong which were developed for PPKTP.

The effective indices of refraction are calculated using the Sellmeier equations given above combined with a tensor transformation, which rotates the inverse dielectric tensor clockwise by an angle *ϕ* as previously defined. Using the commercial software package Comsol Multiphysics and its RF (Radio Frequency) Module [17], we calculate the effective indices of refraction in certain waveguides over a range of orientations. We use the simulation region and diffusion profile from Levine *et al.* [12]. This region consists of a doped-rectangular area of width *w* between two quarter circles of KTP with 20 *μ*m radii and a (40 *μ*m + *w*) × 4 *μ*m rectangular region of air on top. The index profile follows the function erfc(−*Z*/*Z*_0_) [18] as it decreases from the bulk RTP value at the crystal surface, *Z* = 0, to the bulk KTP value within the waveguide, *Z* ≤ 0.

**Fig. 1:**
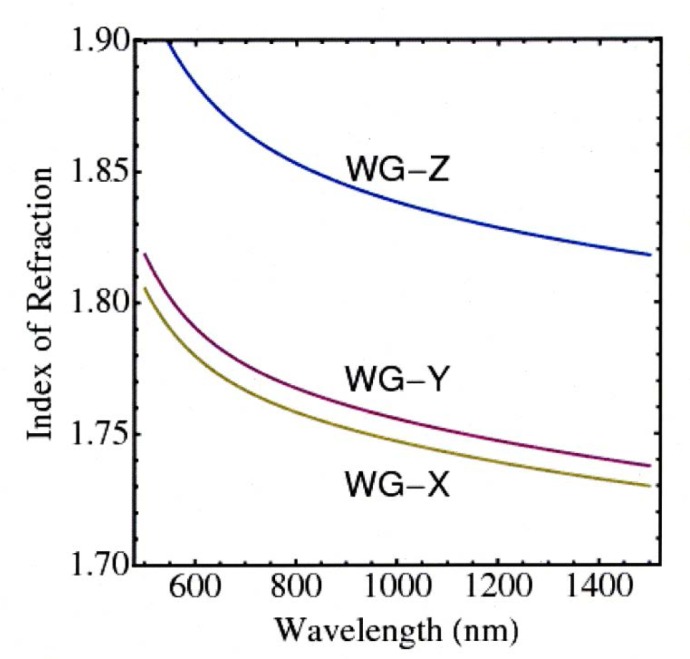
(Color online) The effective indices of refraction for the 3.5 *μ*m × 4.5 *μ*m Rb:KTP waveguide described in the text are given for the three crystal axes.

The effective indices of refraction are shown in Fig. 1. Although the crystal is nearly uniaxial, the splitting between the *X* and *Y* polarizations leads to significant tuning of the operational wavelengths.

## 3. Simulation Results

**Fig. 2:**
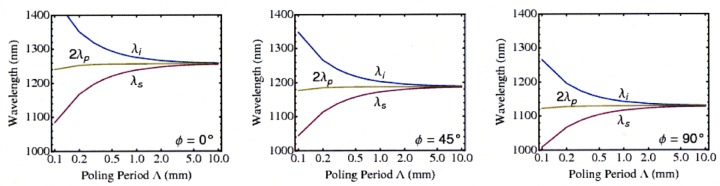
(Color online) The signal, idler, and pump wavelengths (*λ_s_*, *λ_i_*, and *λ_p_*, respectively) as a function of poling period for 3.5 *μ*m × 4.5 *μ*m Rb:KTP waveguide for various orientations. As the orientation angle *ϕ* is increased, the quasi-phase-matched frequency for each poling period decreases. The *ϕ* = 0° case was presented previously [12].

The effective indices of refraction are used to find the phase-matched wavelengths resulting in polarization-entangled type-II SPDC in periodically poled Rb:KTP waveguides. We study a 3.5 *μ*m × 4.5 *μ*m Rb:KTP waveguide, where the dimensions are chosen so that the signal and idler will have a single mode in the waveguide. We find that the degenerate output wavelength decreases from 1257 nm at *ϕ* = 0° to 1130 nm at *ϕ* = 90°, as shown in the right sides of the graphs in Fig. 2. Our operating point involves satisfying both parts of (2), so Fig. 2 differs from a typical tuning curve. The phase-matched frequencies decrease as *ϕ* increases because the crystal *Y* index of refraction is greater than the crystal *X* index of refraction for KTP. The quasi-phase-matched wavelengths for type-II SPDC in bulk KTP and various Rb:KTP waveguides using a poling period of Λ=1 mm are presented in Table 1. Because this study uses collinear propagation, the bulk case is only a theoretical exercise.

To find the effect of angular dependence on efficiency, we calculated the effective nonlinear coefficients for type-II phase matching in KTP given by Ref. [19] where | *d*_15_ |= 1.2 pm/V and | d_24_ |=2.2 pm/V, where a small dispersion correction has been included. An additional factor of 2/*π* is required to account for quasi-phase matching vs. standard phase matching [20]. The effective nonlinearity in the *XY* plane of KTP is given by

(4) $$d_{\mathrm{eoe}}=d_{\mathrm{oee}}=d_{15}\;\sin^{2}\phi +d_{24}\;\cos^{2}\phi .$$

The efficiency may be calculated using Eq. (6) of Ref. [21] enhanced by a factor of 2 to account for the two processes. We choose a crystal length of 36 mm to match Ref. [22] and calculate at quasi-phase matching a maximum rate of 4⋅10^6^ s^−1^ GHz^−1^ mW^−1^ to 1⋅10^6^ s^−1^ GHz^−1^ mW^−1^ as *ϕ* varies from 0° to 90°, with a full-width at half-maximum (FWHM) bandwidth which decreases roughly linearly from 1.3 nm to 0.7 nm in this angular range. This compares to a value of 3⋅10^5^ s^−1^ GHz^−1^ mW^−1^ in a recent experiment using a PPLN waveguide followed by discrete optical components [22], suggesting the present scheme is potentially a competitive entangled photon source.

Fidelity is a measure of the utility of the states for use in two-photon interference experiments and other quantum information applications [1,2]. For example, a fidelity value of *F* > 0.78 is required to satisfy the Clauser-Horne-Shimony-Holt inequality [23,24] which was proposed for a loophole-free version of Bell’s inequality. Such fidelity has been demonstrated experimentally including *F* = 0.82 by Santori *et al.* [25], *F* = 0.99 by Peters *et al.* [26], *F* =0.92 by Politi *et al.* [27], and *F* = 0.97 by Ling *et al.* [28].

**Fig. 3:**
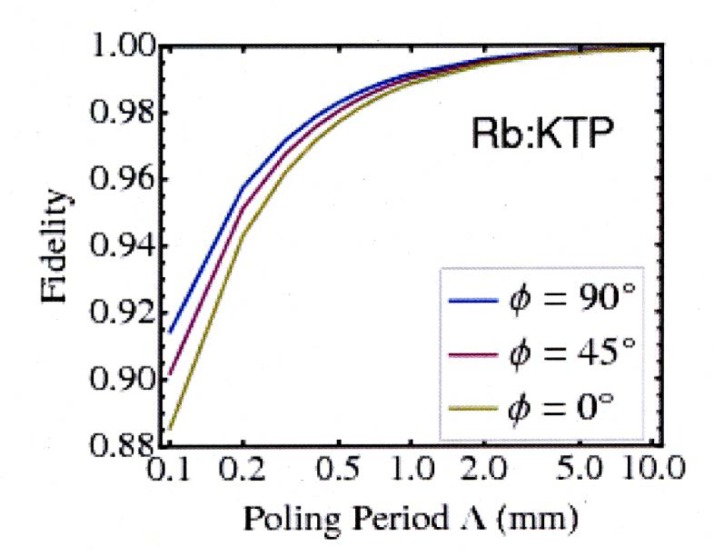
(Color online) Fidelity as a function of poling period for the 3.5 *μ*m × 4.5 *μ*m Rb:KTP waveguide. The *ϕ* = 0° line (bottom curve) for the Rb:KTP waveguide is the result found by Levine *et al.* [12].

Here, we find that across the orientations, the waveguides maintain the high fidelity calculated by Levine *et al.* [12], as shown in Fig. 3. As in the earlier work, the predictions are subject to certain numerical approximations which limit the maximum predicted fidelity to 0.99 ± 0.01 at 1 mm, with increasing uncertainty for the longer poling periods.

## 4. Concluding Remarks

We propose a scheme which allows the generation of entangled photons using type-II SPDC in periodically poled waveguides over a range of frequencies. By orienting the waveguide relative to the crystal axes, we predict a degenerate output wavelength range of 1130 nm to 1257 nm in an unpoled 3.5 *μ*m × 4.5 *μ*m Rb:KTP waveguide. Other high-efficiency operation points may be achieved by using other biaxial materials such as potassium titanyl arsenate (KTA).

The proposed waveguides maintain a moderate efficiency and high fidelity regardless of the orientation of the waveguide relative to the crystal axes. By introducing a new degree of freedom, polarization-entangled type-II SPDC may operate over a broader range of wavelengths.

## Figures and Tables

**Table 1:** Phase-matched signal, idler, and pump wavelengths (*λ_s_*, *λ_i_*, and *λ_p_*, respectively) using a poling period of 1 mm in Rb:KTP waveguides and bulk KTP

*w*(*μ*m)	*Z*_0_ (*μ*m)	*ϕ*	*λ_s_* (nm)	*λ_i_* (nm)	*λ_p_* (nm)
3.5	4.5	0°	1239	1275	628
		45°	1172	1203	594
		90°	1117	1142	565

5.0	6.0	0°	1212	1247	615
		45°	1156	1185	585
		90°	1110	1135	561

bulk KTP		0°	1069	1093	540
		45°	1025	1047	518
		90°	985	1003	497
